# QTL Mapping and Genome-Wide Association Study Reveal Genetic Loci and Candidate Genes Related to Soluble Solids Content in Melon

**DOI:** 10.3390/cimb45090450

**Published:** 2023-08-26

**Authors:** Honglang Yan, Kang Wang, Manman Wang, Lulu Feng, Huimin Zhang, Xiaoyun Wei

**Affiliations:** Jiangsu Yanjiang Institute of Agricultural Sciences, Jiangsu Academy of Agricultural Sciences, Nantong 226012, China; wangkang@jaas.ac.cn (K.W.); manmanwang@jaas.ac.cn (M.W.); fenglulu@jaas.ac.cn (L.F.); zhanghuimin1@jaas.ac.cn (H.Z.); 20042200@jaas.ac.cn (X.W.)

**Keywords:** melon, soluble solids content, linkage analysis, GWAS, candidate genes

## Abstract

Melon (*Cucumis melo* L.) is an economically important Cucurbitaceae crop grown around the globe. The sweetness of melon is a significant factor in fruit quality and consumer appeal, and the soluble solids content (SSC) is a key index of melon sweetness. In this study, 146 recombinant inbred lines (RILs) derived from two oriental melon materials with different levels of sweetness containing 1427 bin markers, and 213 melon accessions containing 1,681,775 single nucleotide polymorphism (SNP) markers were used to identify genomic regions influencing SSC. Linkage mapping detected 10 quantitative trait loci (QTLs) distributed on six chromosomes, seven of which were overlapped with the reported QTLs. A total of 211 significant SNPs were identified by genome-wide association study (GWAS), 138 of which overlapped with the reported QTLs. Two new stable, co-localized regions on chromosome 3 were identified by QTL mapping and GWAS across multiple environments, which explained large phenotypic variance. Five candidate genes related to SSC were identified by QTL mapping, GWAS, and qRT-PCR, two of which were involved in hydrolysis of raffinose and sucrose located in the new stable loci. The other three candidate genes were involved in raffinose synthesis, sugar transport, and production of substrate for sugar synthesis. The genomic regions and candidate genes will be helpful for molecular breeding programs and elucidating the mechanisms of sugar accumulation.

## 1. Introduction

Melon (*Cucumis melo* L.; 2*n* = 2x = 24) is an economically important Cucurbitaceae crop grown around the globe. Based on differences of ovary pubescence, melon cultivars are divided into two subspecies: *C. melo* ssp. *agrestis* and *C. melo* ssp. *melo* [1,2]. The sweetness is an important quality trait of melons that affects consumer preferences, and the soluble solids content (SSC) is a key index of melon sweetness. Identifying genomic regions and genes controlling SSC will be helpful for molecular breeding.

Linkage analysis is an effective approach for detecting quantitative trait loci (QTLs) related to complex agronomic traits using biparental segregating populations, and several QTLs were found to regulate the accumulation of soluble solids content in melon [3,4,5,6,7,8,9,10,11,12,13,14]. However, a considerable number of QTLs for soluble solids content detected through diverse backgrounds have wide intervals, and these QTLs are difficult to apply to marker-assisted selection (MAS). Due to the rapid growth of sequencing technology, GWAS has proven a powerful approach in detecting genetic variations of important agronomic traits in melon [15,16,17,18,19]. Previously, some key loci related to soluble solids content were detected via GWAS in tomato [20,21], apricot [22], peach [23], and watermelon [24]. Leida et al. [25] identified a stable region on chromosome 3 associated with sugar content via association analysis using 251 single nucleotide polymorphism (SNP) markers. Wang et al. [26] detected 11 SNPs associated with soluble solids content in melon via GWAS using genotyping-by-sequencing (GBS).

Association mapping does not require a biparental segregating population and is useful for analyzing multiple alleles [27], but the population substructure and allele frequency could bring about false-positive results [28]. Combining association analysis and linkage analysis has been successfully used for dissecting complex quantitative traits in rice [29], maize [30], and soybean [31]. Wei et al. [29] identified eight candidate genes for rice seedlings in response to high temperature stress using linkage analysis and GWAS. Xu et al. [30] dissected the genetic basis of zinc deficiency tolerance in maize at the seedling stage via linkage mapping and GWAS, and three candidate genes were identified as being responsible for Zn transport. Hu et al. [31] identified a gibberellin 3b-hydroxylase gene related to soybean 100-seed weight through joint linkage mapping and GWAS which enhanced photosynthesis and increased seed yield in soybean. At present, association analysis and linkage analysis have been used to dissect the complex agronomic traits of melon. Nimmakayala et al. [32] detected several QTLs for fruit firmness through GWAS and QTL mapping. Perpiñá et al. [33] detected several stable QTLs related to SSC using linkage analysis with introgression lines and association analysis in backcross populations. Oren et al. [34] detected a major netting QTL on melon chromosome 2 by combining linkage analysis and association analysis. Du et al. [35] identified a candidate genomic region associate with the fruit surface groove of melon via linkage analysis and association analysis. These genetic loci identified by GWAS and linkage analysis will make MAS breeding more accurate and effective. Based on the rapid growth of sequencing technology, joint GWAS and linkage analysis will play an increasingly important role in gene mining for important agronomic traits in melon. Raffinose and stachyose are the core sugars of cucurbit crops transported in the phloem [36]. Based on the rapid advances of molecular biology and genomics, many genes related to sugar metabolism and accumulation during fruit growth have been reported such as alpha-galactosidase (AGA) [37,38], sugars will eventually be exported transporter (SWEET) [37,38,39], vacuolar sugar transporter (VST) [40], sucrose transporter (SUT) [39,41], tonoplast sugar transporter (TST) [42,43,44], hexose transporter [45], tonoplast H+/sugar antiporters [46], trehalose-6-phosphate (T6P) [47,48], sucrose synthase (SUS), sucrose phosphate synthase (SPS), and invertase [49].

Previous studies have shown that the integration of linkage analysis and GWAS provided a higher power and resolution to investigate complex quantitative traits. In the present study, we constructed a recombinant inbred line (RIL) population containing 146 lines derived from two oriental melon materials with different levels of sweetness. Then, a high-resolution genetic map of the RIL population was constructed to detect QTLs related to SSC in melon. In addition, a total of 213 melon accessions were utilized to detect the significance of SNPs associated with SSC via GWAS. Five candidate genes were identified by QTL mapping, GWAS, and qRT-PCR, which played important roles in synthesis and hydrolysis of raffinose, sucrose hydrolysis, and sugar transport. The genomic regions and candidate genes will be helpful for molecular breeding programs and elucidating the mechanisms of sugar accumulation.

## 2. Materials and Methods

### 2.1. Plant Materials and Phenotypic Data Analysis

The plant material contained an RIL population and a GWAS panel. The RIL population, consisting of 146 RILs, was derived from two oriental melon cultivars (1214 with low sweetness and 1228 with high sweetness). The RIL population was grown in a greenhouse at Xueyao Experiment Station (32°14′ N, 120°64′ E) of Jiangsu Yanjiang Institute of Agricultural Sciences (Nantong, China) in the spring of 2020, 2021, and 2022. The GWAS panel, consisting of 213 melon accessions that included 145 accessions of ssp. *agrestis*, 67 accessions of ssp. *melo*, and 1 Mapao melon accession, was planted in a greenhouse at Xueyao Experiment Station in the spring of 2020 and 2021 and the spring/autumn of 2021. A randomized block design with three replicates was utilized to evaluate the plant materials. Five plants were grown for each melon germplasm. One fruit per plant was harvested at 30–35 days after pollination, and the SSC of fruit flesh was measured with a hand-held digital PAL-1 refractometer (Atago). ANOVA for SSC was calculated in R using the aov function. Based on the phenotypic data of two populations for multiple environments, the best linear unbiased predictions (BLUP) were calculated by the R package lme4.

### 2.2. DNA Extraction and Whole-Genome Resequencing

The young leaves of RIL population and GWAS panel were utilized to extract DNA using the CTAB method. The genomic DNA samples were fragmented to a size of 350 bp for sequencing on the Illumina HiSeq PE150 platform of Novogene, China. The melon reference genome HS_ZJU [50] and its annotation were downloaded online (http://www.ncbi.nlm.nih.gov, accessed on 19 July 2021). BWA [51] was utilized to map the clean reads to the melon reference genome using the command ‘mem -t 4 -k 32 -M’. SNP calling was performed using SAMtools [52] with a Bayesian approach. The high-quality SNPs were performed using Genome Analysis Toolkit (GATK) software 4.0.4.0 [53]. ANNOVAR software (Version 20130520) [54] was utilized to annotate the SNPs identified in this study.

### 2.3. Linkage Analysis

The sequencing depths of parents (1214 and 1228) and RILs were 20-fold and 5-fold, respectively. The RILs were genotyped by polymorphic SNPs with an aa × bb segregation pattern between the parental materials. The recombinant breakpoints were evaluated by the sliding window approach [55]. The genotype was performed with a sliding window of 15 SNPs. The high-resolution genetic map of the RIL population was constructed with bin markers using JoinMap 4.1 (https://www.kyazma.nl/index.php/JoinMap/, accessed on 13 May 2022). The ALLMAPS program [56] was utilized to visualize the relationships between the physical and genetic locations. The composite interval mapping (CIM) method of Windows QTL Cartographer 2.5 software (https://brcwebportal.cos.ncsu.edu/qtlcart/WQTLCart.htm, accessed on 7 June 2022) was utilized to detect QTLs. The permutation test of MapQTL6.0 (https://www.kyazma.nl/index.php/MapQTL/, accessed on 7 June 2022) was utilized to calculate the LOD threshold values for the SSC.

### 2.4. Association Analysis

The sequencing depths of 211 melon accessions were 10-fold, and two accessions (1214 and 1228) were sequenced with a depth of 20-fold. The sequencing depth ≥ 6, missing rate ≤ 0.5, and minor allele frequency ≥ 0.05 were considered standard in identifying SNPs. Finally, a total of 1,681,775 SNPs were used for association analysis. The software TreeBeST 1.9.2 (https://treesoft.sourceforge.net/treebest.shtml, accessed on 2 April 2022) was utilized to create an individual-based neighbor-joining (NJ) tree using p-distance with 1000 bootstrap replications. The phylogenetic tree was visualized using MEGA6.0 (http://www.megasoftware.net/, accessed on 2 April 2022). The program ADMIXTURE (version 1.23) [57] was utilized to calculate the population genetics structure using an expectation maximization algorithm. To evaluate the LD decay in the association population, the software PopLDdecay 3.40 [58] was utilized to calculate the degree of linkage disequilibrium coefficient (*r*^2^) between pairwise SNPs with the command ‘-n -dprime -minMAF 0.05’. The GEMMA 0.98.1 software package [59] was utilized to perform association analysis, and the significant P-value threshold was approximately 1 × 10^−6^.

### 2.5. Candidate Genes Identification and qRT-PCR Analysis

The co-detected regions by linkage analysis and GWAS, and the ±100 kb regions around the significant SNPs located within the reported QTL regions were considered significant regions to search for candidate genes. Annotations of these genes were analyzed following the melon reference genome ‘HS_ZJU’, and genes related to sugar metabolism and accumulation were considered candidate genes. The fruits of the two parents (1228 and 1214) harvested at 20DAP, 25DAP, and 30DAP were used to extract RNA for qRT-PCR analysis. The cDNA was synthesized using All-in-One First-Strand cDNA Synthesis SuperMix for qPCR (TransGen, Beijing, China). Gene expression was performed on the ABI7500 Real-Time PCR System (Applied Biosystems, Singapore) using Green qPCR SuperMix (TransGen, Beijing, China). The data of relative gene expression was analyzed according to the 2^−ΔΔCt^ method with the 18s rRNA used as the internal control. The primers used in this study are shown in Table S8.

## 3. Results

### 3.1. Phenotypic Variation of Soluble Solids Content in Two Populations

In this study, 146 RILs and 213 melon accessions were used. The SSC of RILs was evaluated in the Nantong greenhouse in the spring of 2020, 2021, and 2022 (Table S9). The SSC of the 213 melon accessions was evaluated in the spring of 2020 and 2022 and the spring/autumn of 2021 (Table S10). The descriptive statistics including the mean, range, standard deviation, kurtosis, skewness, and coefficient of variation (CV) for SSC of two panels are presented in Table 1.

**Figure 1:**
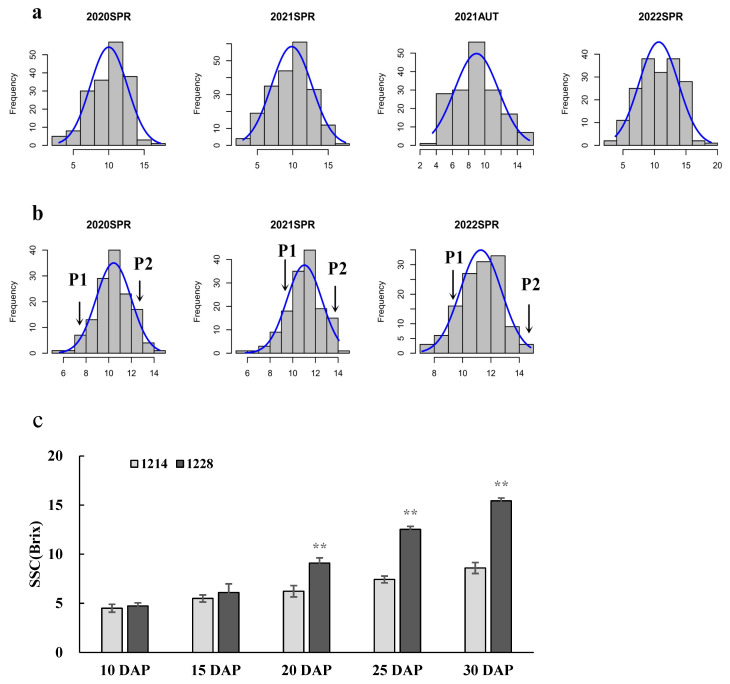
Frequency distribution and changes in soluble solids content. (**a**) Frequency distribution of SSC for 213 melon accessions in four environments. (**b**) Frequency distribution of SSC for RILs in three environments. SPR represents spring and AUT represents autumn. Arrows indicate the soluble solids content for the parents, 1214(P1) and 1228(P2). (**c**) SSC in the fruit during the five ripening stages. ** Significant at *p* ≤ 0.01.

The SSC of RILs ranged from 5.67% to 14.50% (with a mean of 10.43%) in spring 2020, from 5.83% to 14.07% (with a mean of 11.01%) in spring 2021, and from 7.15% to 14.80% (with a mean of 11.29%) in spring 2022. The SSC of 213 melon accessions ranged from 3.00% to 17.30% (with a mean of 10.01%) in spring 2020, from 3.00% to 17.43% (with a mean of 9.86%) in spring 2021, from 3.55% to 15.50% (with a mean of 8.97%) in autumn 2021, and from 3.80% to 18.98% (with a mean of 10.68%) in spring 2022. The CV of the RILs ranged from 12.93% to 14.96%, while the CV of the accessions ranged from 26.17% to 30.21%. The skewness and kurtosis in Table 1 and the histograms in Figure 1a,b show that the data of SSC have a normal distribution. The melon accessions (G), environment (E), and the genotype × environment interaction (G × E) had a significant effect (*p* < 0.01) on the SSC (Table 1). The broad-sense heritability (*H*^2^) for SSC were 0.65 and 0.72 in two populations (Table 1).

The fruits of the two parents (1228 and 1214), were harvested at five growth stages to investigate the changes in the SSC (Figure 1c). With fruit development, the SSC of 1228 and 1214 accumulated continuously and peaked at 30 days after pollination (DAP), but the SSC of 1214 was significantly lower than that of 1228 at each stage (Figure 1c). The different levels of SSC led to the differences in sweetness between these two cultivars.

### 3.2. Population Sequencing and Linkage Map Construction

**Figure 2:**
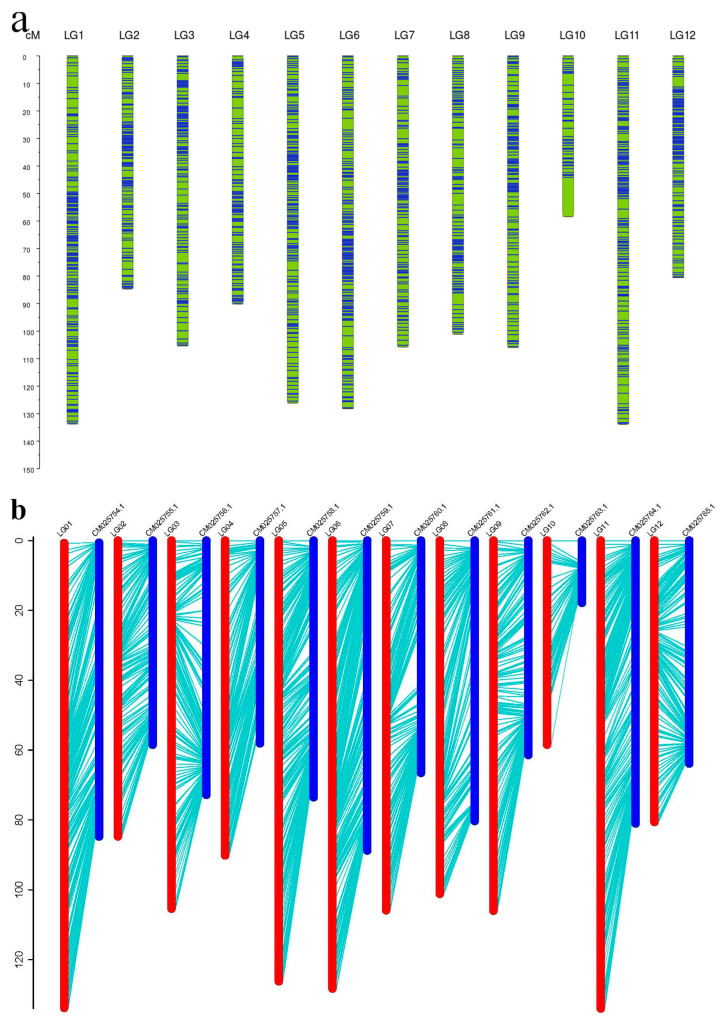
Construction of the linkage map (**a**) and collinearity analysis between the linkage map and melon genome ‘HS_ZJU’ (**b**).

In order to construct a high-resolution genetic map, 146 RILs together with two parents were re-sequenced on the Illumina HiSeq PE150 platform. In total, 9.05 Gb (19.96-fold genome coverage) and 10.34 Gb (23.04-fold genome coverage) clean bases were obtained for two parents (1214 and 1228), respectively (Table S1). A total of 364 Gb clean bases were obtained for the 146 RILs with high quality (Q20 ≥ 95.42%, Q30 ≥ 88.72%), and the average sequencing depth for each individual was 6.7-fold (Table S1). A total of 380,864 SNPs were detected between the parents, and 111,500 SNPs were identified with the aa×bb segregation pattern. Then, a total of 1427 bin markers were obtained to construct a high-resolution genetic map (Figure 2a). The total length of the bin map was 1254.34 cM, and the average interval between the adjacent markers was 0.88 cM (Table 2). The genetic and physical positions of the bin markers on the 12 chromosomes were compared with each other, and a high level of collinearity was detected between the linkage map and the reference genome of melon cultivar ‘HS_ZJU’ (Figure 2b). However, there were several chromosomal locations of the bins that displayed inconsistencies with the genetic map.

### 3.3. Identification of QTLs for Soluble Solids Content

**Figure 3:**
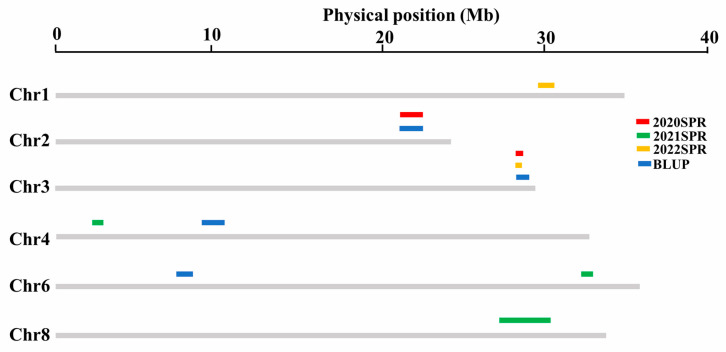
Distribution of QTLs related to SSC on the melon chromosomes. Gray bars represent the chromosomes and colored bars represent the QTLs for SSC. Bar length indicates the physical distance of QTLs.

Under three growth environments and BLUP, 14 QTLs related to SSC were identified using Windows QTL Cartographer 2.5 software with the bin map. These 14 QTLs were scattered on chromosomes 1, 2, 3, 4, 6 and 8, as follows: 1 QTL on chromosome 1, 4 QTLs on chromosome 2, 4 QTLs on chromosome 3, 2 QTLs on chromosome 4, 2 QTLs on chromosome 6, and 1 QTL on chromosome 8 (Figure 3, Table S3). The physical distances of these QTLs ranged from 0.39 to 3.15 Mb, and the average distances was 0.88 Mb. The LOD value of these QTLs ranged from 3.2 to 5.7, and the phenotypic variation explained (PVE) values ranged from 6.7% to 13.8%. All the QTL showed negative additive effects, indicating that alleles from the high-SSC parent 1228 contributed to the greater SSC. Based on the physical position of flanking markers, the 14 QTLs were clustered into 10 common QTLs. Of the 10 common QTLs, one QTL was repeatedly detected across three environments, and two QTLs were detected across two environments. In addition, seven common QTLs were mapped to the same loci of previously reported QTLs (Table S3) and confirmed the reliability of the genetic map.

### 3.4. GWAS for Soluble Solids Content

**Figure 4:**
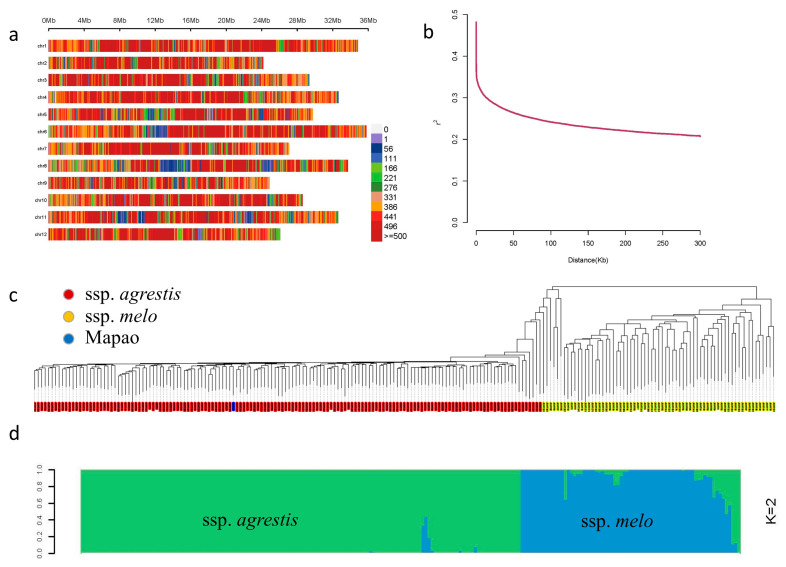
Distribution of the SNPs and the population structure of 213 melon accessions. (**a**) Distribution of SNPs and nucleotide diversity across the melon genome ‘HS_ZJU’. Different colors indicate the number of SNPs within the 0.1 Mb window size. (**b**) Genome-wide average LD decay for 213 melon accessions. (**c**) Phylogenetic tree of 213 melon accessions. (**d**) Population structure of melon accessions estimated by ADMIXTURE with k = 2.

We re-sequenced 213 melon accessions at an average sequencing depth of 9.7× on the Illumina HiSeq PE150 platform, generating 941.6 Gb resequencing data of high quality (Q20 ≥ 94.43%, Q30 ≥ 86.92%, Table S2). The raw sequence data reported in this paper have been deposited in the Genome Sequence Archive at the National Genomics Data Center, China National Center for Bioinformation/Beijing Institute of Genomics, Chinese Academy of Sciences, under accession number CRA011939 and are publicly accessible at https://ngdc.cncb.ac.cn/gsa (released on 24 July 2023). The resequencing data were mapped to the melon genome ‘HS_ZJU’, and 1,681,775 high-confidence SNPs (missing rate ≤ 0.5; minor allele frequency ≥ 0.05) were detected. Of these, 99,547, 51,623, 304,931, 1,029,614, and 96,114 SNPs were located in upstream regions, exons, introns, intergenic regions, and downstream regions, respectively (Table S4). The markers were scattered on the melon genome, with the lowest (107,238) and highest (174,458) number of markers present on chromosome 9 and chromosome 1, respectively (Figure 4a). Additionally, the highest SNP frequency was 5.04 SNPs/kb on chromosome 7. The linkage disequilibrium (LD) level was calculated as the physical distance at which *r*^2^ decreased to half of the maximum value, and LD decay was estimated at 103 kb (Figure 4b). From the phylogenetic tree constructed with the high-confidence SNPs (Figure 4c), the 213 accessions were divided into two subpopulations: subpopulation I primarily consisted of ssp. *agrestis* and subpopulation II consisted of ssp. *melo*. The result was further supported by a population structure plot (Figure 4d). In addition, the ‘Mapao’ melon accession was located within ssp. *agrestis*, and this result was consistent with a previous study [17].

**Figure 5:**
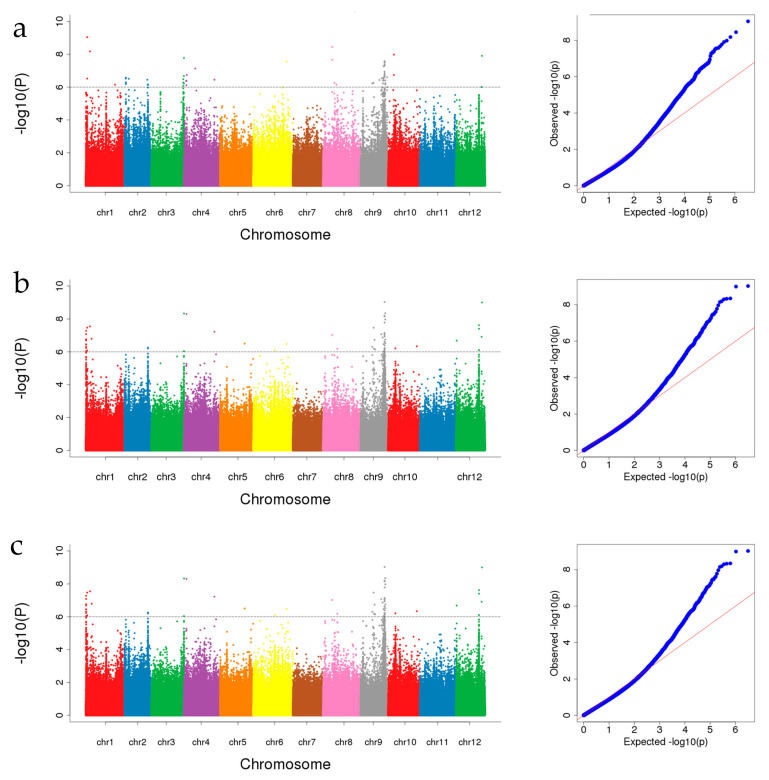

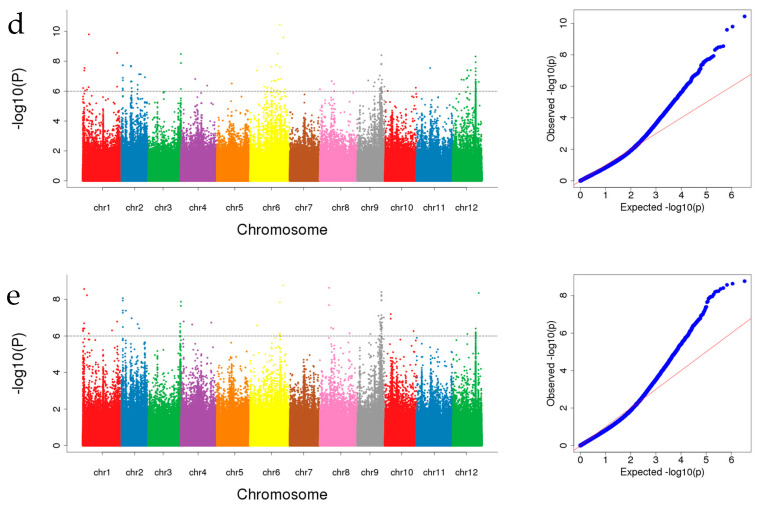
GWAS signals for SSC estimated across four environments and BLUP. Manhattan plot and quantile–quantile (Q–Q) plot estimated in (**a**) spring 2020 (E1), (**b**) spring 2021 (E2), (**c**) autumn 2021 (E3), (**d**) spring 2022 (E4), and (**e**) BLUP. The gray lines on the *Y*-axis denote the significance threshold (−log10 *p* > 6).

**Figure 6:**
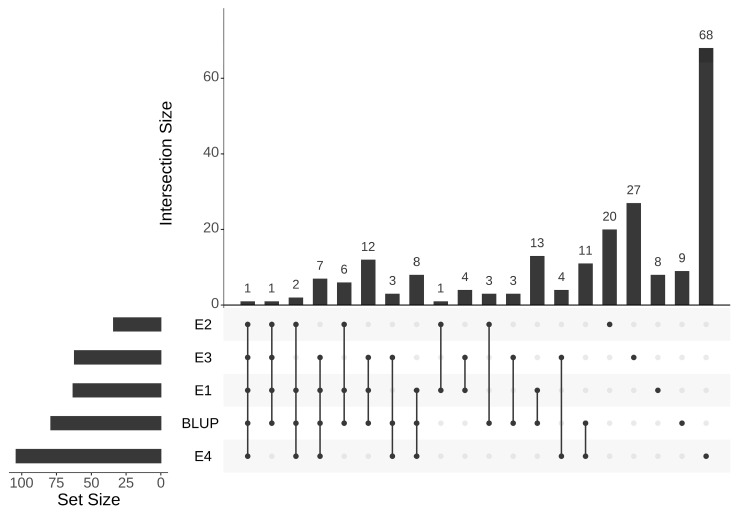
Intersection of significant SNPs across four different environments (E1, E2, E3, and E4) and BLUP.

Based on the data of four growth environments and BLUP, GWAS of 213 melon accessions was performed with 1,681,775 SNPs using GEMMA software. At a significance level of *p* < 10^−6^, a total of 211 SNPs significant associated with SSC was found (Figure 5; Table S5). Among these SNPs, s6-26722606 had the lowest *p*-value (−log10 *p* = 10.44), which was detected in 2022SPR. The two chromosomes with the highest number of markers were chromosome 9 with 61 SNPs and chromosome 12 with 34 SNPs. The intersections based on the SNPs detected across four environments and BLUP are presented in Figure 6 as the UpSet plot [60]. The UpSet plot showed the number of significant markers detected in each environment and BLUP, and also showed the number of the same markers detected across different environments (Figure 6). In Figure 6, 29 significant SNPs were detected coherently across three environments, 10 significant SNPs were detected coherently across four environments, and 1 significant SNP was detected coherently across all environments. Compared to the SNP loci detected by GWAS and the QTLs detected in previous studies, 138 SNPs overlapped with the reported QTLs (Table S6).

### 3.5. Co-Detected Regions by QTL Mapping and GWAS

Linkage analysis and association analysis could be complementary methods for QTLs detection. The results of linkage analysis and GWAS were analyzed together to identify stable regions significantly associated with SSC. Five QTLs identified by linkage analysis and 13 SNPs identified by GWAS were co-localized on chromosome 3, 4, and 6 (Table 3). QTL *qSSC3-1* and five SNPs, and *qSSC3-2* and four SNPs were co-localized on chromosome 3. QTL *qSSC4-1* and one SNPs, and *qSSC4-2* and two SNPs were co-localized on chromosome 4. QTL *qSSC6-2* and one SNP were co-localized on chromosome 6. The two co-localized regions on chromosome 3 were identified across multiple environments with a large PVE (>10%) and likely included genes affecting the accumulation of SSC.

### 3.6. Identification of Candidate Genes Related to SSC

The genes located within QTL intervals and the LD regions of significant SNPs, which were related to sugar metabolism and accumulation, were considered as candidate genes. As a result of *qSSC3-1* and *qSSC3-2* being co-detected by linkage analysis and GWAS across multiple environments, candidate genes related to SSC were searched for in these two stable regions on chromosome 3. A total of 113 genes were detected within the two co-localized intervals (28.5–28.8 Mb and 28.9–29.3 Mb) on chromosome 3, which consisted of 46 and 67 genes, respectively (Table S7). Of the 46 genes in *qSSC3-1*, the invertase gene *CWINV3* (*MELO 06759*) may be a candidate gene for SSC as a key enzyme for converting sucrose into glucose and fructose. The expression of *CWINV3* was significantly different between the high sweetness and low sweetness oriental melon accessions at different fruit growth stages [61]. Meanwhile, we found an alpha-galactosidase (*MELO 06810*) in *qSSC3-2*, which was related to the production of galactose and sucrose during phloem unloading [62]. Yang et al. [50] reported that *MELO 06810* had a parallel shift of amino acid in Cucurbitaceae via positive selection gene analysis, which may play an important role in phloem transport.

**Figure 7:**
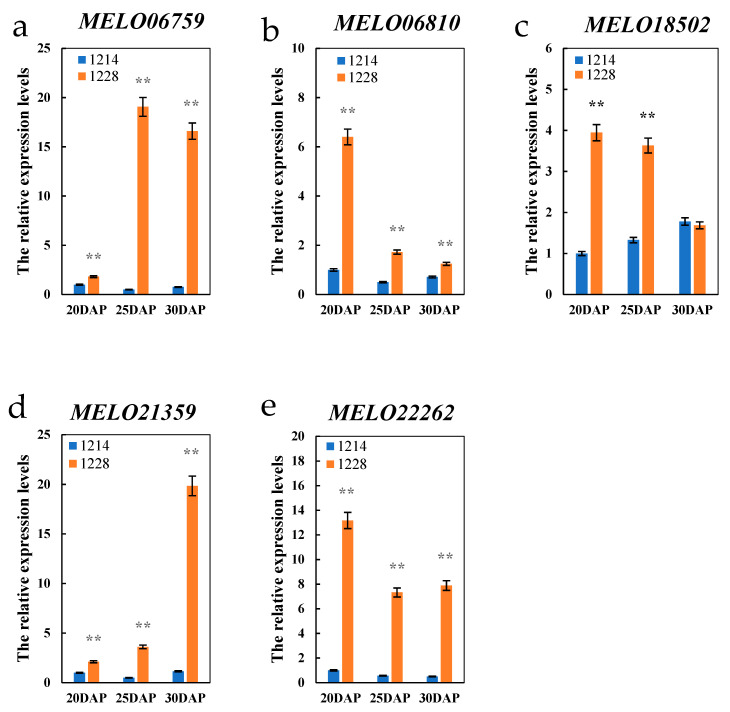
Relative expression levels of five candidate genes. (**a**) *MELO06759*; (**b**) *MELO06810*; (**c**) *MELO18502*; (**d**) *MELO21359*; and (**e**) *MELO22262*; ** Significant at *p* ≤ 0.01.

Based on the LD decay of 103 kb (Figure 4b), the ±100 kb regions around 138 significant SNPs that overlapped with the reported QTLs (Table S6) were considered significant regions to search for candidate genes. In these regions, nine candidate genes, which included sucrose phosphate synthase (SPS), trehalose 6-phosphate phosphatase (TPP), raffinose synthase (RS), sucrose synthase (SS), UDP-glucose 4-epimerase (UGE), sugar transport, and sugars will eventually be exported transporter (SWEET), were identified as being related to sugar metabolism and accumulation (Table 4). Quantitative real-time PCR was used to estimate the relative expression of 11 candidate genes at the stage of SSC rapid accumulation (Figure 1c). The results showed that five candidate genes had different expression levels between the two parents at the stage of 20DAP, 25DAP, and 30DAP (Figure 7). The expression levels of *MELO21359*, *MELO06759*, *MELO22262*, and *MELO06810* were significantly higher in 1228 than 1214 at three stages. *MELO18502* had higher expression levels in 1228 than 1214 at 20DAP and 25DAP, while no significant difference was observed at 30DAP. *MELO22262* and *MELO06810* shared a pattern of higher expression level at 20DAP, and decreased expression at 25DAP and 30DAP. *MELO21359* had the higher expression level at 30DAP, while *MELO06759* had the higher expression levels at 25DAP and 30DAP. These five genes with different expression levels between two parents are the presumed candidates controlling SSC in melon.

## 4. Discussion

### 4.1. Phenotypic Data over Multiple Years and High-Density Markers Improved the Accuracy of QTL Mapping and Gwas

Linkage mapping based on biparental segregating populations and GWAS based on a natural population are complementary approaches to detect genetic variations for important agronomic traits. Linkage mapping can only evaluate limited variations between two parents in a single population. However, Association analysis can investigate all the allelic variations in natural populations. For QTL mapping and GWAS, phenotypic data over multiple years could reduce environmental factors to identify genetic loci that consistently affect the trait. The sweetness of melon has a significant effect on its quality and consumer appeal, and SSC is an important index of melon sweetness. In this study, phenotypic data of the two melon populations were investigated for three years. In addition, two cultivars with different SSC levels were utilized to understand the accumulation process. We found that SSC accumulated quickly from 20 DAP to 30 DAP, and a similar accumulation pattern for sugar has been observed in previous studies [50,61]. Higher marker density could also enhance the accuracy of linkage analysis and GWAS. In this study, 1427 bin markers generated via genome resequencing were used to construct a high-resolution genetic linkage map with an average interval between the adjacent markers of 0.88 cM. For GWAS, 1.68 million high-confidence SNPs were used in this study, leading to a high precision of GWAS to investigate the complex traits.

### 4.2. QTL Intervals and SNP Loci for SSC Identified via Linkage Mapping and GWAS

In this study, 10 QTLs (with an average distance of about 0.88 Mb) related to SSC were identified by linkage mapping using the high-resolution genetic linkage map. Seven QTLs were mapped to the same loci of previously reported QTLs (Table S3) and confirmed the reliability of our study. Based on genome resequencing, 1.68 million marks were utilized to detect the loci related to SSC via GWAS. A total of 211 SNPs significantly associated with SSC were found by GWAS with a significance level of *p* < 10^−6^. Compared to the SNP loci detected by GWAS and the QTLs detected in previous studies, 138 SNPs overlapped with the reported QTLs (Table S6). These results indicate that the loci identified in the present study were reliable for candidate genes mining.

In this study, we found that 5 QTLs detected by linkage analysis were co-localized with 13 significant SNPs detected by GWAS. The genomic region overlapped by *qSSC4-2*, S4_10141036, and S4_10275303 on chromosome 4, and *qSSC6-2* and S6_32656543 on chromosome 6 (Table 3, Table S3 and Table S6) were also identified within the QTLs identified in previous studies [4,10]. Due to limited parental materials used in QTL mapping, not all of the genetic variations in melon have been identified, and populations derived from different materials, as in previous studies, could determine new QTLs or confirm previous QTLs [8]. In previous studies, the high sweetness parental materials utilized to construct segregated populations were almost always muskmelon. Saladie et al. [63] showed that the patterns of sugar accumulation were different between the oriental melon and muskmelon during fruit development. In this study, two oriental melon inbreeds with different soluble solids content were utilized to derive a segregated population for QTL mapping, and two stable QTLs, *qSSC3-1* and *qSSC3-2*, identified across multiple environments might be new QTLs associated with SSC.

### 4.3. Further Analysis of Candidate Genes Differentially Expressed between Parents at Different Fruit Growth Stages

Comparing the QTL intervals and SNP loci identified by linkage analysis and GWAS, the co-detected loci and ±100 kb regions around the significant SNPs within the reported QTLs were considered reliable regions to search for candidate genes. According to the annotation information, the genes related to sugar metabolism and accumulation were considered candidate gene controlling soluble solids content. The relative expression levels of eleven genes predicted to participate in the accumulation of SSC were investigated by qRT-PCR at different fruit stages. Finally, five genes with different expression levels between two parents during different stages were considered putative candidates controlling SSC.

In sweet melon, sucrose, fructose, and glucose are the major soluble sugars, and sucrose is the main factor affecting total sugar content [19,49,64]. The cell wall invertases (CWINV) hydrolyzing sucrose into fructose and glucose play an important role in sugar accumulation [65]. Zanor et al. [66] reported that silencing the cell wall invertase LIN5 could lead to a linear decrease in the total soluble solids content in tomato. Wang et al. [45] showed that a reduction in *LIN5* activity caused a decrease of fructose, glucose, and SSC in tomato. A previous study revealed that the expression of *CWINV3* was significantly higher in the high sweetness rather than low sweetness oriental melon accessions at different fruit growth stages [61]. In the present study, the same expression pattern of *CWINV3* was observed (Figure 7a) and the result indicated that *CWINV3* might be involved in SSC accumulation.

Alpha-galactosidases (AGA) that hydrolyze stachyose and raffinose play an important role in mediating source–sink communication during fruit development [62,67,68]. A previous study showed that *ClAGA2* controls fruit raffinose hydrolysis in watermelon, and that the sucrose content of fruits increased significantly in the overexpressed plants [67]. Based on the positive selection analysis in Cucurbitaceae genomes including *C. lanatus*, *C. maxima*, *C. pepo*, *C. sativus*, *L. siceraria*, and *C. melo* (HS, DHL92, Payzawat) with *A thaliana*, *S. lycopersicum*, *M. domestica*, and *O. sativa*, a parallel shift of an amino acid in alpha-galactosidase (*MELO 06810*) was identified in Cucurbitaceae, which may play an important role in raffinose hydrolysis [50]. In this study, the expression of *MELO 06810* was significantly higher in 1228 than 1214 at different fruit growth stages (Figure 7b). Therefore, the alpha-galactosidase (*MELO 06810*) located in *qSSC3-2* is considered a candidate gene related to SSC accumulation.

Raffinose could be hydrolyzed into sucrose and then transported and stored in tissues or cells. Raffinose synthase is a key enzyme for raffinose production, and overexpression of raffinose synthase significantly increased raffinose content [69]. A previous study revealed that the expression of raffinose synthase (*MELO18502*) was significantly higher in the high sweetness rather than low sweetness oriental melon accessions at different fruit growth stages [61]. In this study, the expression of *MELO18502* was significantly higher in 1228 than 1214 at 20DAP and 25DAP(Figure 7c).

Additionally, UDP-glucose 4′-epimerase (UGE) could catalyze the interconversion of UDP-glucose and UDP-galactose, and UDP-glucose may further serve as a substrate for the synthesis of sucrose. A previous study reported that overexpression of UGE could improve the accumulation soluble sugars [70]. In this study, the expression of *MELO21359* was significantly higher in 1228 than 1214 at three stages (Figure 7d).

Sugar transport proteins (STP) played an important role on sugar accumulation via the transport of glucose from the apoplast into plant cells [39,71]. A previous study showed that the soluble solids content increased in the overexpressed plants [45]. In this study, the expression of *MELO22262* was significantly higher in 1228 than 1214 at three stages (Figure 7e).

Sugar metabolism and accumulation are complex biological processes that require the synergistic action of key genes. In this study, five candidate genes related to SSC were identified by QTL mapping, GWAS, and qRT-PCR. These five genes play important roles in the synthesis and hydrolysis of raffinose, sucrose hydrolysis, sugar transport, and production of substrate for sugar synthesis. High gene expression levels of these five genes may collectively promote sugar accumulation.

## 5. Conclusions

The sweetness of melon is a significant factor in fruit quality and consumer appeal, and SSC is a key index of melon sweetness. In this study, a recombinant inbred population including 146 lines derived from two oriental melon materials with different sweetness levels and an association population consisting of 213 melon accessions were utilized to detect genomic regions influencing SSC. By comparing the results of linkage analysis and GWAS, we identified seven QTLs and 138 significant SNPs overlapped with the reported QTLs. In addition, we identified two new stable loci on chromosome 3 via QTL mapping and GWAS across multiple environments. Five candidate genes related to SSC were identified by QTL mapping, GWAS, and qRT-PCR, two of which were involved in raffinose and sucrose hydrolysis located in the new stable loci. The other three candidate genes were involved in raffinose synthesis sugar transport, and production of substrate for sugar synthesis. Synergistic action of these five candidate genes may cause the higher SSC. The identification of genomic regions and candidate genes that influence SSC will provide useful information and insights for molecular breeding and offer an opportunity for sugar accumulation research.

## Figures and Tables

**Table 1:** Descriptive statistics and variance analysis for SSC of the two panels in multiple environments.

Population	Environment ^a^	Parents		Mean ± SE (%)	SD ^b^	Range (%)	CV ^c^ (%)	Skewness	Kurtosis	G ^d^	E ^d^	G × E ^d^	*H* ^2 e^
		1214	1228										
RILs	2020 SPR	7.43	12.40	10.43 ± 0.13	1.56	5.67–14.50	14.96	−0.15	0.13	**	**	**	0.65
	2021 SPR	9.26	13.48	11.01 ± 0.13	1.55	5.83–14.07	14.07	−0.35	0.46				
	2022 SPR	9.62	14.35	11.29 ± 0.13	1.46	7.15–14.80	12.93	−0.34	−0.01				
Accessions	2020 SPR	-	-	10.01 ± 0.20	2.62	3.00–17.30	26.17	−0.39	−0.17	**	**	**	0.72
	2021 SPR	-	-	9.86 ± 0.20	2.87	3.00–17.43	29.01	−0.17	−0.57				
	2021 AUT	-	-	8.97 ± 0.21	2.71	3.55–15.50	30.21	0.16	−0.57				
	2022 SPR	-	-	10.68 ± 0.23	3.12	3.80–18.98	29.21	−0.03	−0.75				

^a^ SPR represents spring and AUT represents autumn; ^b^ SD represents the standard deviation; ^c^ CV represents the coefficient of variation; ^d^ G, E, and G × E represent the effect for the genotype, environment, and genotype × environment interaction, respectively; ^e^ broad-sense heritability. ** Significant at *p* ≤ 0.01.

**Table 2:** Information of the novel constructed genetic linkage map for melon.

LGs	Number of Bins	Map Length (cM)	Average Distance (cM)	Max Gap (cM)	<5 cM	>5 cM
LG01	148	133.75	0.90	4.27	148	0
LG02	109	84.73	0.78	3.00	109	0
LG03	119	105.39	0.89	3.84	119	0
LG04	96	90.16	0.94	3.84	96	0
LG05	152	126.17	0.83	3.42	152	0
LG06	158	128.21	0.81	4.27	158	0
LG07	114	105.84	0.93	3.84	114	0
LG08	109	101.16	0.93	4.27	109	0
LG09	118	105.97	0.90	3.00	118	0
LG10	43	58.46	1.36	14.26	42	1
LG11	149	133.92	0.90	3.84	149	0
LG12	112	80.59	0.72	3.84	112	0
total	1427	1254.34	0.88	14.26	1426	1

**Table 3:** Co-detected loci by QTL mapping and GWAS.

Chr.	Environment	LOD	Position Interval (cM)	Physical Position (bp)	SNP Loci	−log10(*p*)				
						2020SPR	2021SPR	2021AUT	2022SPR	BLUP
3	2020SPR	4.5	95.2–104.3	28,509,689–28,965,705	28,672,844					6.01
3	2022SPR	5.5	93.7–99.5	28,446,470–28,845,998	28,694,392	6.27	6.05			6.27
3	BLUP	5.7	95.6–102.9	28,509,689–28,947,998	28,711,496	6.47				6.51
					28,714,701					6.19
					28,724,776	6.08				6.67
3	BLUP	5.2	102.9–105.1	28,947,998–29,341,849	29,267,782	6.68		6.04	8.49	7.65
					29,283,426				6.14	
					29,289,157	6.22				
					29,330,788	7.77		8.33	7.88	7.88
4	2021SPR	3.5	20.9–28.6	2,334,788–3,020,226	2,491,434	6.74				6.80
4	BLUP	3.4	51.7–54.2	8,961,531–10,360,279	10,141,036	7.13				6.63
					10,275,303		7.06			
6	2021SPR	3.5	95.5–101	32,217,543–32,935,488	32,656,543		6.56			

**Table 4:** Candidate genes related to SSC within the QTL/SNP regions.

Gene	Chromosome	Start	End	Functional Annotation
*MELO03698*	chr2	14,397,615	14,406,002	Sucrose-phosphate synthase
*MELO06759*	chr3	28,826,329	28,829,358	Beta-fructofuranosidase, insoluble isoenzyme CWINV3
*MELO06810*	chr3	29,119,436	29,126,262	Alpha-galactosidase
*MELO12562*	chr6	6,109,512	6,114,363	Trehalose 6-phosphate phosphatase
*MELO18502*	chr8	16,852,497	16,859,087	Raffinose synthase
*MELO20547*	chr9	14,794,455	14,800,212	Sucrose synthase
*MELO21359*	chr9	23,772,893	23,777,340	UDP-glucose 4′-epimerase
*MELO22262*	chr10	5,145,518	5,147,793	Sugar transport protein 10-like
*MELO27354*	chr12	20,598,706	20,606,584	Trehalose-6-phosphate synthase
*MELO27634*	chr12	23,004,024	23,006,015	Sugar transporter SWEET12-like
*MELO27635*	chr12	23,013,800	23,015,623	Sugar transporter SWEET12-like

## Data Availability

The datasets analyzed in this study are available in the manuscript text and Supplementary Materials. The raw sequence data reported in this paper have been deposited in the Genome Sequence Archive at the National Genomics Data Center, China National Center for Bioinformation/Beijing Institute of Genomics, Chinese Academy of Sciences, under accession number CRA011939 and are publicly accessible at https://ngdc.cncb.ac.cn/gsa (released on 24 July 2023).

## Supplementary Materials

The following supporting information can be downloaded at: https://www.mdpi.com/article/10.3390/cimb45090450/s1, Table S1: The sequence data summary for RILs; Table S2: Sequence information on the genomes of 213 melon accessions; Table S3: QTL analyses of soluble solids content in melon; Table S4: Results of filtered SNP annotation; Table S5: The SNPs with significant association signals (−log10(*p*) ≥ 6) for SSC; Table S6: GWAS signals overlapped with the reported QTLs; Table S7: Annotations of the genes underlying *qSSC3-1*, and *qSSC3-2* in melon; Table S8: Primer sequences for qRT-PCR analysis; Table S9: Phenotypic data of SSC for RILs; Table S10: Phenotypic data of SSC for the accessions.
